# Dysbiosis, Host Metabolism, and Non-communicable Diseases: Trialogue in the Inborn Errors of Metabolism

**DOI:** 10.3389/fphys.2021.716520

**Published:** 2021-09-06

**Authors:** Chiara Montanari, Sara Parolisi, Elisa Borghi, Lorenza Putignani, Giulia Bassanini, Juri Zuvadelli, Cristina Bonfanti, Albina Tummolo, Carlo Dionisi Vici, Giacomo Biasucci, Alberto Burlina, Maria Teresa Carbone, Elvira Verduci

**Affiliations:** ^1^Department of Pediatrics, Vittore Buzzi Children’s Hospital, University of Milan, Milan, Italy; ^2^UOS Metabolic and Rare Diseases, AORN Santobono, Naples, Italy; ^3^Department of Health Science, University of Milan, Milan, Italy; ^4^Department of Diagnostic and Laboratory Medicine, Unit of Microbiology and Diagnostic Immunology, Unit of Microbiomics and Multimodal Laboratory Medicine Research Area, Unit of Human Microbiome, Bambino Gesù Children’s Hospital, IRCCS, Rome, Italy; ^5^Clinical Department of Pediatrics, ASST Santi Paolo e Carlo, San Paolo Hospital, University of Milan, Milan, Italy; ^6^Rare Metabolic Disease Unit, Pediatric Department, Fondazione MBBM, San Gerardo Hospital, Monza, Italy; ^7^Metabolic Diseases and Clinical Genetics Unit, Children’s Hospital Giovanni XXIII, Bari, Italy; ^8^Division of Metabolism, Bambino Gesù Children’s Hospital, Rome, Italy; ^9^Department of Paediatrics & Neonatology, Guglielmo da Saliceto Hospital, Piacenza, Italy; ^10^Division of Inborn Metabolic Diseases, Department of Diagnostic Services, University Hospital of Padua, Padua, Italy

**Keywords:** microbiota, inborn errors of metabolism, diet, gut-liver axis, non-communicable diseases, enterophenotype

## Abstract

Inborn errors of metabolism (IEMs) represent a complex system model, in need of a shift of approach exploring the main factors mediating the regulation of the system, internal or external and overcoming the traditional concept of biochemical and genetic defects. In this context, among the established factors influencing the metabolic flux, i.e., diet, lifestyle, antibiotics, xenobiotics, infectious agents, also the individual gut microbiota should be considered. A healthy gut microbiota contributes in maintaining human health by providing unique metabolic functions to the human host. Many patients with IEMs are on special diets, the main treatment for these diseases. Hence, IEMs represent a good model to evaluate how specific dietary patterns, in terms of macronutrients composition and quality of nutrients, can be related to a characteristic microbiota associated with a specific clinical phenotype (“enterophenotype”). In the present review, we aim at reporting the possible links existing between dysbiosis, a condition reported in IEMs patients, and a pro-inflammatory status, through an altered “gut-liver” cross-talk network and a major oxidative stress, with a repercussion on the health status of the patient, increasing the risk of non-communicable diseases (NCDs). On this basis, more attention should be paid to the nutritional status assessment and the clinical and biochemical signs of possible onset of comorbidities, with the goal of improving the long-term wellbeing in IEMs. A balanced intestinal ecosystem has been shown to positively contribute to patient health and its perturbation may influence the clinical spectrum of individuals with IEMs. For this, reaching eubiosis through the improvement of the quality of dietary products and mixtures, the use of pre-, pro- and postbiotics, could represent both a preventive and therapeutic strategy in these complex diseases.

## Introduction

The gut microbiota takes an active part in the flux and in the disease phenotype (Putignani et al., 2019) and can be influenced, especially in pediatric life, by many factors, including genetic variation, diet, lifestyle, antibiotics, xenobiotics, and infectious agents (Putignani et al., 2014; Lynch and Pedersen, 2016). The host-associated gut microbiota, which is composed of around 10–100 trillion microorganisms (Turnbaugh et al., 2007), contributes to maintaining human health by providing unique metabolic functions to the human host (Zhang et al., 2000). Functional maturation of the gut microbiota takes place during the first 3years of life (Han, 2014; Lynch and Pedersen, 2016) and plays an important role in extracting energy from food fibers and complex carbohydrates that are not digested in the upper portion of the human digestive tract (Gill et al., 2006). The gut microbiota is also involved in stimulating and promoting the maturation of the immune system and in facilitating the resistance against pathogens (Martin et al., 2013; Sommer and Bäckhed, 2013; Lynch and Pedersen, 2016). It interacts with the host by influencing immunity, metabolism, health status (Tremaroli and Backhed, 2012) and the association between a wide range of diseases and dysbiosis has long been well known (Guinane and Cotter, 2013).

Inborn errors of metabolism (IEMs) represent a complex system model, mainly due to genetic mutations affecting a single gene but which are also influenced by different determinants, both internal and external, contributing to the disease phenotype (McCabe, 2019). External factors such as diet, lifestyle, and antibiotics are the main environmental constraints regulating human metabolism.

A novel-challenge to approach IEMs is to consider the metabolic flux as part of a dynamic system, moving from a static concept of biochemical and genetic defects to a novel way. The key point is to evaluate the metabolic flux in a real-time manner to assess the actual metabolome dynamics. Omics sciences (e.g., genomics, metagenomics, transcriptomics, proteomics, metaproteomics, metabolomics, meta-metabolomics, and interactomics) allow to stratify in a system biology approach, related-multi-omics data within layers describing the entire process of disease phenotyping (Lanpher et al., 2006; Putignani et al., 2016).

The cornerstone therapy of many IEMs is dietary treatment, based on strict regimens that must be maintained lifelong, therefore making this disease category an ideal model to evaluate how specific dietary patterns can be related to a characteristic hallmark of the microbiota (“enterophenotype”). Interestingly, recent studies have shown a high rate in the variability of the intestinal ecosystem in relation to different dietary patterns (Putignani and Dallapiccola, 2016), making the use of advanced technologies applied to microbiota study an effective approach to define the “core” bacteria profiles associated with different dietary patterns (Harmsen et al., 2000; Zimmer et al., 2012). Diets excessively rich or poor in some nutrients, a common characteristic of dietary intervention in several IEMs, can promote a possible state of dysbiosis with potential systemic effects (Colonetti et al., 2018). Considering that the gut microbiota may influence some specific clinical phenotypes, this paper aims at exploring the possible links between IEMs, with external modulating factors, with the gut microbial community, and the health status of affected individuals (Reijngoud, 2018), also in relation to the eventual presence of non-communicable diseases (NCDs). Moreover, we want to discuss the potential contribution of gut microbiota in the challenging research of new treatments for these complex diseases.

## Dietary Treatment in Iems and Gut Microbial Profile

Few studies available in the current literature report the composition of gut microbiota in IEMs, in particular in phenylketonuria (PKU) and glycogen storage disease (GSD) in relation to diet composition.

In PKU (OMIM261600), an inherited metabolic disease determined by the impaired activity of the enzyme phenylalanine hydroxylase, with a consequent phenylalanine (Phe) accumulation in the blood, a special “low phe intake” diet should be started as early as possible in the first weeks of life to hamper neurological damages (Singh et al., 2014; van Spronsen et al., 2017). Dietary treatment consists of three pillars: (i) natural protein restriction, (ii) Phe-free-l-amino acid supplements, and (iii) low protein foods. Indeed, the special low protein products, which are variants/substitutes of some common “natural” foods, such as bread pasta), are rich in sugars to ameliorate the palatability, determining a daily glycemic index and glycemic load in PKU patients higher than the normal values (Moretti et al., 2017). Phe-restricted diet shows similarity with the Mediterranean diet -high vegetable and fiber intake- that has been reported to promote growth of beneficial bacteria and the increase of their derived metabolites (Tosti et al., 2018).

However, the quality of carbohydrates intake is a key factor in determining the gut microbial composition and the short-chain fatty acid (SCFA) production, since different bacteria possess different metabolic pathways to break down sugars (Hamaker and Tuncil, 2014). To date, few studies have analyzed the intestinal microbiota in subjects with PKU, and data are summarized in Supplementary Table S1 in supplemental materials.

Verduci et al. (2018) highlighted in PKU children a low gut microbial biodiversity, generally considered the first distinctive sign of intestinal dysbiosis (Mosca et al., 2016). Moreover, shifts in microbiota composition lead to a different microbial metabolite production, mainly affecting the SCFAs profile (Scott et al., 2013). The most relevant differences between PKU and mild hyperphenylalaninemia (MHP) children microbiota concern the Firmicutes phylum, with a consequent reduction of total fecal SCFAs and butyrate. Furthermore, the PKU gut microbiota was enriched in the genera *Blautia* and *Clostridium* and depleted in the genus *Faecalibacterium* as well as in *Lactobacillus* spp. (Pinheiro de Oliveira et al., 2016; Bassanini et al., 2019). Also in adult PKU patients, the gut microbiota, recently investigated by Mancilla et al. (2021), showed a similar pattern with an enrichment in *Clostridium* and a depletion in *Faecalibacterium*, but with a less pronounced abundance of *Blautia* spp.

Glycogen storage diseases are hereditary metabolic disorders caused by the deficiency of enzymes involved in glycogen metabolism. In particular, GSD Ia and Ib, are hepatic GSDs due to defects in the glucose-6-phosphatase complex, sharing hepatomegaly and fasting hypoglycemia as the main clinical abnormalities. The aim of treatment is to prevent hypoglycemia and secondary metabolic complications (Kishnani et al., 2014). Dietary therapy is based on frequent meals, with a high intake of complex carbohydrates, restriction of sugars, and a lower amount of lipids compared to the general population. Part of carbohydrates is supplied by uncooked cornstarch (UCCS) that patients take at regular intervals several times a day, and also during the night, to maintain euglycemia (Heller et al., 2008; Kishnani et al., 2014). Given the peculiarity of the GSD-I diet, an impact on gut microbiota can be expected. Indeed, a GSD-linked gut dysbiosis has been recently reported in two works, summarized in Supplementary Table S2 in supplemental materials. The main reported characteristics in GSD patients are a reduction in intestinal microbiota richness and differences in taxa relative abundances compared with the control group, with a dramatic increase in Proteobacteria at the expense of Firmicutes and Bacteroidetes, the two main phyla generally colonizing healthy subjects’ gut microbiota (Colonetti et al., 2019; Ceccarani et al., 2020). Furthermore, the beneficial genera *Faecalibacterium* and *Oscillospira* were significantly reduced, while the Proteobacteria abundance was characterized by a high presence of *Enterobacteriaceae* family, within which *Escherichia* spp. was found significantly increased, with a pro-inflammatory activity capable of further triggering intestinal imbalance. At metabolic level, SCFAs quantification showed a significant increase of fecal acetate and propionate in GSD subjects (Ceccarani et al., 2020). Based on these findings, gut dysbiosis might contribute to several comorbidities of the GSD phenotype, including obesity, inflammatory bowel disease (IBD), and chronic liver disease. Other GSD patients, like GSD-III patients, may require a hyperproteic and hyperlipidic diet, similar to a ketogenic diet, with a lower use of UCCS. However, no sufficient data about the microbiota composition are available neither in GSD-III patients nor in other GSDs types (Ceccarani et al., 2020).

## Management-Related Factors That (Potentially) Affect the Gut Microbiota in Iems

As previously described, the bioavailability of dietary substrates affects the gut microbial composition and leads to a different production of microbial metabolites (Verduci et al., 2018). Beyond the dietary pattern, the type and the quality of specific nutrients used in the treatment of IEMs can be a potential influencing factor able to further modify the gut microbiota composition.

Starting from the few available studies, we have summarized in Table 1 the possible alterations of the gut microbiota related to (i) the use of special dietary products, such as low-protein foods (LPs; Wang et al., 2019) and protein substitutes (Davila et al., 2013; Sawin et al., 2015; Lin et al., 2017; van Wegberg, 2017; Yang and Liao, 2019), (ii) the use of specific nutrients, such as UCCS (Kishnani et al., 2014; Colonetti et al., 2019), medium chain triglycerides (MCTs; Bach and Babayan, 1982; Decuypere and Dierick, 2003; Das et al., 2010; Zentek et al., 2013), and long-chain polyunsaturated fatty acids (LC-PUFAs; Pinheiro de Oliveira et al., 2016; Pu et al., 2016; Costantini et al., 2017; Watson et al., 2018; Bassanini et al., 2019), or (iii) the exclusion/reduction of specific nutrients, as breast milk (Coppa et al., 2006; MacDonald et al., 2006; Hascoet et al., 2011; Videhult and West, 2016) and (iv) the utilization of special nutrition mode, as tube feeding (O’Keefe, 2010; Takeshita et al., 2011; Burlina et al., 2018).

**Table 1:** Potential effects of dietary factors on gut microbiota in IEMs.

Dietary factors	Potential effects on microbiota
**(i) Special dietary products**	
Low-protein foods (LPs)	Low protein foods are starches that constitute the major intake of carbohydrates in patients with amino acid metabolism diseases: - enriched in sugars: ↑ glycemic Index and glycemic load → reduction of SCFAs production inm phenylketonuria (PKU) patients (Moretti et al., 2017; Verduci et al., 2018); - variable proportion of amylose and amylopectin (not known) and a variable content of soluble fiber. Starches with a high amylose content and soluble fiber could increase the production of SCFAs (Wang et al., 2019).
Protein substitutes	In patients with aminoacidopathies, most of the protein requirement is guaranteed by free amino acids (free-AAs) formulas. AAs act as a supplemental substrate for SCFA production by the gut microbiota; AAs metabolites impact the gut epithelia physiology, both in a beneficial (e.g., butyrate and indole) and deleterious (e.g., ammonia) way (Davila et al., 2013; Lin et al., 2017); Glutamate increases microbial diversity and promote gut colonization by *Faecalibacterium prausnitzii* and *Roseburia* (Yang and Liao, 2019) In PKU treatment, an alternative of Phe-free-AA supplements can be represented by the glycomacropeptide -GMP-, a low-Phe protein source derived from whey protein (van Wegberg et al., 2017). GMP supplementation → anti-inflammatory effect, ↓ *Desulfovibrio* spp.; ↑ production of SCFAs (Sawin et al., 2015)
**(ii) Specific nutrients**	
Uncooked cornstarch (UCCS)	UCCS is normally used in the dietetic treatment of glycogen storage disease (GSD) I for maintaining blood glucose concentration in the desirable range (Kishnani et al., 2014). UCCS’s overload in GSD type I patients→ reduction of the fecal pH with alteration in the relative abundances of fermenting bacteria species and reduction of SCFAs (Colonetti et al., 2019)
Medium chain triglycerides (MCTs)	MCTs are widely used in the dietary treatment of some metabolic diseases, like in long-chain fatty acid (LCFA) oxidation disorders or in the deficiency of the carnitine system (Bach and Babayan, 1982), as well as in the ketogenic diet and recently also in GSDs (Das et al., 2010). In animal models MCTs prevent LPS-mediated endotoxemia improving the intestinal barrier integrity and affecting the gut microbial composition in terms of Gram-positive and Gram-negative ratio (Decuypere and Dierick, 2003) In weaning piglets, a supplementation of medium-chain fatty acids (MCFAs) seems to increase the abundance of *Escherichia*, *Hafnia*, *Shigella* and *Clostridia* (Zentek et al., 2013)
Long-chain Polyunsaturated Fatty Acids (LC-PUFAs)	Early supplementation with LC-PUFAs and DHA is strongly recommended in PKU patients (Giovannini et al., 2012). PUFAs are able to restore a eubiotic status, after dysbiosis, in animal model; increase the production of SCFAs acting as prebiotics for Bacteroidetes and butyrate-producing bacteria belonging to the Lachnospiraceae family (Costantini et al., 2017) A mixed DHA/EPA supplement on healthy subjects seems to: ↑ *Clostridiaceae*, *Sutterellaceae* and *Akkermansiaceae* ↑ *Bifidobacterium* ssp. and *Oscillospira* spp. ↓ *Coprococcus* spp. and *Faecalibacterium* spp. (Watson et al., 2018) DHA-enriched high oleic canola oil supplementation in individuals at risk for metabolic syndrome seems to: ↓ *Faecalibacterium* spp. (Pu et al., 2016). These changes is common observed in phenylketonuric cohorts that are supplemented with DHA patients (Pinheiro de Oliveira et al., 2016; Bassanini et al., 2019)
**(iii) Exclusion/reduction of specific nutrients**	
Lower consumption of breast milk	Early dietetic intervention in IEMs often consists in the total exclusion of breastfeeding from the diet (e.g., in galactosaemia) or in a limited intake in most diseases (MacDonald et al., 2006). → reduction of *Bifidobacteria* and *Lactobacillus* (Coppa et al., 2006; Hascoet et al., 2011; Videhult and West, 2016)
**(iv) Special nutrient feeding**	
Tube feeding	Tube feeding is commonly used as a treatment strategy for children with methylmalonic acidemia (MMA) and propionic acidemia (PA; Burlina et al., 2018) Enteral tube feeding can disrupt the oral indigenous microbiota (Takeshita et al., 2011) and in PA/MMA creates a permissive environment for *Clostridioides difficile* infection (O’Keefe, 2010); With enteral tube feeding, there is also the difficulty to maintain an adequate fluid intake → possible constipation

Chronic use of drugs in IEM patients may represent another important factor influencing the composition of the gut microbiota. The disease itself, or specific comorbidities related or not to metabolic control, can require pharmacological treatment. It is documented that drug-induced shifts in the composition of gut microbiota can be also associated with disease progression (Sayers et al., 2018). To date, many studies investigated the role of drugs, especially antibiotics, on gut microbiota but few data have been collected on this aspect in IEMs. We summarized in Table 2 the possible microbiota profile modifications in relation to the use of specific medications, as allopurinol (Kishnani et al., 2014; Yu et al., 2018; Ceccarani et al., 2020), antibiotics (metronidazole; Baumgartner et al., 2014; Jakobsson et al., 2010) and the supplementation with l-carnitine (Tang et al., 2013; Wilson et al., 2016; Chen et al., 2019; Vernocchi et al., 2020).

**Table 2:** Potential effects of use of specific medications on the gut microbiota in IEMs.

Medication	Potential effects on microbiota
Allopurinol	Gout can be a long-term complication in GSDs patients related to the high levels of uric acid. Although a good metabolic control may prevent hyperuricemia, allopurinol treatment is recommended to prevent gout attacks (Kishnani et al., 2014). In hyperuricemic rates was observed: ↑ *Bifidobacterium* and *Collinsella* ↓ *Adlercreutzia* and *Anaerostipes* (Yu et al., 2018) Microbiota of GSD patients taking allopurinol showed a slight increase of *Bifidobacterium* (Ceccarani et al., 2020)
Metronidazole	In PA/MMA an option to reduce intestinal propionate synthesis is the administration of metronidazole (25% of propionate is produced in the gut by anaerobic fermentation; Baumgartner et al., 2014) → reduction of species biodiversity and total bacterial population (long term risk of drug resistance; Jakobsson et al., 2010)
L-carnitine	L-carnitine is commonly used in many IEMs to balance the primary or secondary carnitine deficiency. It is metabolized into trimethylamine (TMA) by several gut microbes, and converted in the liver to trimethylamine N-oxides (TMAO; Tang et al., 2013; Chen et al., 2019; Vernocchi et al., 2020), that decreases the transport of reverse cholesterol and bile acid synthesis → possible atherosclerosis mediator (Koeth et al., 2013; Wilson et al., 2016)

## The Role of Gut Immune System in Inflammation

A dynamic interplay between the host and his gut microbiota seems to be a key point to achieve and maintain the immune homeostasis. Providing pivotal molecular signals through microbial surface antigens and host metabolites, a consequent maturation of immune tissues and a coordinated immune response take place (Rooks and Garrett, 2016). A functional characterization of microbiome is nowadays possible by means of the high-throughput DNA sequencing technologies. Indeed, these methods have paved the way for many descriptive studies on microbial ecology and have better investigated the interaction between microbial metabolism and host development. Since the gut microbiota plays a crucial role in the host immune system, alterations in its composition and function can trigger a large number of physiological processes, such as low-grade inflammation and excess lipid accumulation, contributing to the development of metabolic perturbations. The patho-mechanisms of inflammatory diseases are still under intense investigation but it seems clear that gut microbiota changes could be involved in the onset and can influence the severity of disease, through a different crosstalk between the host and its microbiota (Webb et al., 2016). Recently, the role of single species in regulating inflammation stage and metabolic disfunctions has been based on complete genome sequencing derived from shotgun metagenomic data that allow characterizing, at species- and strain-level, the enteric microbial communities and key metabolic features (Gophna et al., 2017). Indeed, the effects of microbiota on the pathogenesis of the “metabolic syndrome/disease” are not only related to the specific microorganisms’ composition, but also to the microbial molecules produced. Specifically, microbial fermentation and production of SCFAs has been shown to be different in some metabolic disorders, with a different potential role in immune system modulation (Webb et al., 2016). SCFAs are produced by the saccharolytic fermentation of non-digestible carbohydrates that escape digestion and absorption in the small intestine, and they are mainly represented by acetate, propionate and butyrate, followed by formate and lactate. Minor amounts of branched chain fatty acids (BCFAs), such as iso-butyrate and iso-valerate, are produced through the protein-derived branched chain amino acids (BCAAs; Russell et al., 2011). SCFAs represent an important source of energy for both intestinal epithelial cells and gut microbiota itself, and maintain the mucosal immunity by fortifying the intestinal epithelium functions. SCFAs also play a crucial role on host immune system functions. They act as histone deacetylase inhibitors (Waldecker et al., 2008), promoting an anti-inflammatory cell phenotype and the immune homeostasis, and they function on G protein-coupled receptors (Brown et al., 2003). The gut microbiota also produces aryl hydrocarbon receptor (AHR) ligands and polyamines (Rooks and Garrett, 2016). The transcription factor AHR has a confirmed role in xenobiotic metabolism and in regulating mucosal immune responses. Li et al. (2011) demonstrated in mice that an increased severity of dextran sodium sulfate-induced colonic inflammation was observed in the absence of AHR ligands while, by supplementing mice diet with synthetic AHR ligands, they achieved an attenuation of the inflammatory status. Indeed, polyamines are primarily involved in the maintenance of intestinal barrier integrity by producing intercellular junction proteins, and many studies have highlighted their role in regulating immune responses (Zhang et al., 2000), and in modulating systemic and mucosal adaptive immunity (Minois et al., 2011). Moreover, the gut microbiota releases formylated peptides that act on G-protein-linked surface receptors, which are expressed on neutrophils and macrophages that recognize gut microbial products and stimulate reactive oxygen species (ROS) production responsible for an increase in cell oxidative stress (Marciano and Vajro, 2017).

## The Cross-Talk Between Gut and Liver

During the past decade, it has become clear that microbial ecology can be involved in the development of a pro-oxidative and pro-inflammatory state (Luca et al., 2019). Oxidative stress along with chronic inflammatory conditions pave the way for the development of several metabolic diseases such as obesity and insulin resistance (Wellen and Hotamisligil, 2005; Heilbronn and Campbell, 2008; Cani and Delzenne, 2009; Pedersen et al., 2016; Rani et al., 2016).

**Figure 1:**
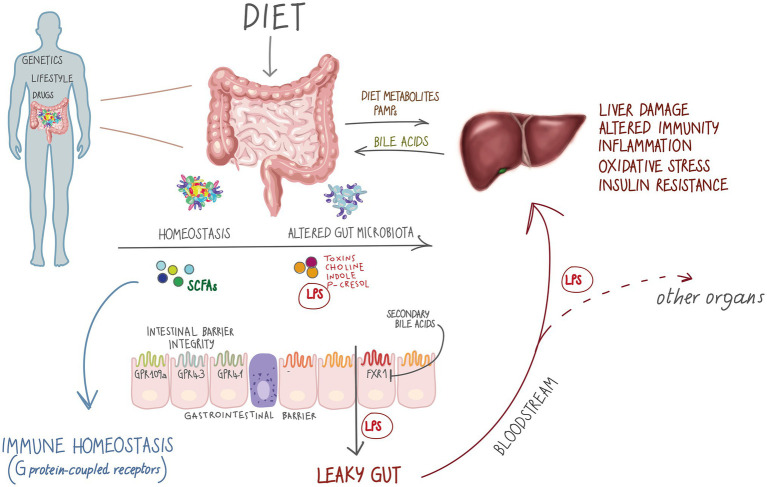
The gut-liver axis and metabolic diseases. Metabolic diseases affect the gut-liver axis through exposome factors such as diet, xenobiotics, sedentary lifestyle as well as genetic treats. On the other hand, in inborn errors of metabolism (IEMs), special diets represent an important modulator of microbiota, impacting the gut-liver axis by altering microbial metabolites production. short-chain fatty acids (SCFAs) interact with metabolite-sensing G protein-coupled receptors GPR4, GPR43 and GPR109A promoting gut homeostasis (Macia et al., 2015) that has a central role in many human body functional axes. Alterations in the microbial community might promote a leaky gut condition, by altering gut barrier permeability and allowing translocation of whole bacteria and metabolites, such as lipopolysaccharide (LPS), further affecting the gut-liver axis and participating in non-communicable disease development.

The ability of the gut microbiota to affect gastrointestinal homeostasis, predisposing to chronic inflammation and modulating other metabolic functions of the host, is mediated by the gut-liver axis (Nieuwdorp et al., 2014). The liver and the intestine talk and influence each other through a bi-directional communication (Figure 1), mediated by biliary tract, portal vein and systemic mediators (Tripathi et al., 2018; Albillos et al., 2020). Bile acids (BAs) and other bioactive factors produced by the liver affect the gut microbiota by controlling unrestricted bacterial overgrowth and maintaining intestinal eubiosis *via* nuclear receptors such as farnesoid X receptor (FXR) and G protein coupled bile acid receptor (GPBAR1; also known as TGR5; Inagaki et al., 2006; Albillos et al., 2020).

On the other hand, the metabolites produced by the host and by the intestinal bioreactor do not limit their effect on the intestinal wall, but, moving through the portal vein, affect the liver and other organs and systems by entering into the circulation (Stärkel and Schnabl, 2016; Tripathi et al., 2018). This dual exchange operates in healthy conditions and plays a fundamental role in pathological contexts (Grenham et al., 2011).

Dysbiosis has been demonstrated to favor oxidative stress and to affect the immunological and inflammatory status of the host (Luca et al., 2019). In particular, it is considered the major driver of increased intestinal permeability (Henao-Mejia et al., 2012; Leclercq et al., 2012). Impaired integrity of gut barrier shifts BAs and choline metabolism, with translocation of microorganisms and their metabolites to the liver through the portal system, resulting in increased circulating levels of lipopolysaccharide (LPS; Ridlon et al., 2014; Mouzaki et al., 2016; Arab et al., 2017). Bacterial LPS endotoxins act as important signaling molecules that trigger and maintain oxidative stress and a low-tone inflammatory state and, by activating pattern recognition receptors such as Toll-like receptors (TLR), causing an innate immune response, insulin resistance and liver damage, thus transferability of disease *via* the microbiota (Uesugi et al., 2001; Cani and Delzenne, 2007; Csak et al., 2011; Anand et al., 2016).

Cani et al. (2007) defined the inflammatory state often associated with metabolic syndrome like “metabolic endotoxemia.” This systemic inflammation is thought to occur as bacteria translocate through the gastrointestinal tract, increasing circulating levels of LPS (Tripathi et al., 2018). Higher circulating levels of LPS have been observed in obese subjects with insulin resistance compared with matched controls (Karlsson et al., 2013). An important link between gut microbiota, intestinal permeability and inflammation is represented by *Akkermansia muciniphila*, a Gram-negative anaerobe whose reduced abundance has been associated with compromising gut barrier integrity, increased inflammation and with both alcoholic and nonalcoholic liver damage (Everard et al., 2013; Borgo et al., 2017; Grander et al., 2018). Among other factors, SCFAs might also modulate the immune response by reducing intestinal permeability (Donohoe et al., 2011). An inadequate SCFAs production by gut microbiota leads to increased intestinal permeability, exacerbating metabolic endotoxemia and subsequent low-grade inflammation. This evidence is also supported by the observation of Everard et al. (2013): administration of propionate-producing bacteria *A. muciniphila* is able to partially reverse the obesity-associated low-grade of inflammation and subsequent insulin resistance induced by translocation of LPS from the intestine to the portal vein in mice, without affecting food intake.

## Does Iem’s Microbiota Share Characteristics with Gut Microbiota of Inflammatory Diseases?

In this section, we aim at comparing the different profiles and at describing the possible pro-inflammatory profile of gut microbiota in PKU and in GSD, mainly in GSD-Ia and GSD-Ib, exploiting inflammatory-related known signatures by decision support systems (DSSs). However, evidence of intestinal inflammation has not been demonstrated in PKU patients and thus it is not listed amongst the characteristic clinical features of the disease.

On the other hand, a dysbiosis in phenylketonuric patients has been recorded, showing some characteristics shared with an inflamed gut microbiota. An increase in pro-inflammatory genera, such as *Escherichia* and *Blautia*, and a depletion in beneficial genera, such as *Faecalibacterium*, *Roseburia*, and *Akkermansia* represent the microbial profile observed in PKU patients. Indeed, *Escherichia* spp. is a Gram-negative microorganism, displaying the LPS on the outer membrane (Hotamisligil, 2006; Bassanini et al., 2019), while *Blautia* stimulates pro-inflammatory cytokines secretion by host cells (Tuovinen et al., 2013). Pinheiro de Oliveira et al. (2016) highlighted a PKU microbiome involvement in LPS biosynthesis, suggesting a potential role of the gut microbiota in promoting gut inflammation.

*Faecalibacterium* is considered a biomarker of intestinal wellness due to its activity in anti-inflammatory molecules production, specifically mediated by the inhibition of the NF-κB pathway (Bassanini et al., 2019), and by SCFAs production. Therefore, the decrease in *Faecalibacterium* abundance and in other beneficial bacteria is associated with low amounts of total SCFAs and, in particular, butyrate (Verduci et al., 2018; Bassanini et al., 2019).

The functional predictive analysis of PKU microbiota suggest how some bacterial functions were underrepresented with a potential influence on starch and sucrose metabolism, glycolysis/gluconeogenesis, as well as biosynthesis of some amino acids, while the overrepresented taxa in PKU subjects were involved in LPS biosynthesis. The functional prediction seems to support the existing correlation between the dietary pattern in PKU, the gut microbiota composition, and the altered glucose metabolism, which in turn envisage a potential influence on the overweight status and obesity onset, promoted in a pro-inflammatory milieu (Verduci et al., 2020).

The gut microbiota in GSD patients has been poorly investigated so far (Colonetti et al., 2019; Ceccarani et al., 2020), and seems to be characterized by a significantly lower biodiversity compared with healthy controls that could indicate a first marker of inflammation. Both studies also showed an enrichment in Proteobacteria and, in particular, *Enterobacteriaceae*, a finding consistent with the dysbiosis reported in IBD patients. The two GSD-I subtypes differ for some clinical features. GSD-Ib patients typically develop neutrophil dysfunction, predisposing them to IBD. Although limited to a very few cases, IBD was recently reported also in GSD-Ia (Lawrence et al., 2017; Carnero-Gregorio et al., 2019), highlighting a potential contribution of GSD-I associated gut microbiota abnormalities in the development of intestinal inflammation. Indeed, *Enterobacteriaceae* exert pro-inflammatory activity both locally, at the gastrointestinal mucosa level, and systemically in genetically predisposed individuals, as GSD-Ib patients. Colonetti et al. (2019) also found an increased *Escherichia/Shigella* abundance. Moreover, the Veillonellaceae family was increased both in GSD patients (Ceccarani et al., 2020) and patients with IBD, probably participating in the pro-inflammatory status. Another pro-inflammatory characteristic in GSD cohorts is determined by the *Faecalibacterium* and *Oscillospira* genera depletion. *Faecalibacterium* spp., as mentioned above, has the ability to produce anti-inflammatory metabolites and to reduce the severity of chemically-induced inflammation in murine models (Sokol et al., 2008; Martin et al., 2014). This genus is considered one of the main butyrate producers found in the intestine (Barcenilla et al., 2000; Duncan et al., 2002). Butyrate can reduce intestinal mucosa inflammation by inhibiting NF-κB transcription factor activation (Inan et al., 2000), upregulating PPARγ (Schwab et al., 2007) and inhibiting interferon gamma release (Klampfer et al., 2003). Therefore, both GSD and PKU certainly share the characteristics of an inflamed intestine, with a potential pro-inflammatory role of their microbiota, then more or less evident clinical manifestations.

## Iems and Non-Communicable Diseases: What’s the Role of Microbiome?

Non-communicable diseases are mainly represented by cardiovascular diseases (heart impairment, stroke, etc.) and diabetes as a consequence of a “metabolic syndrome,” characterized by overweight/obesity, hypertension, raised blood sugar, insulin resistance and raised cholesterol (World Health Organization, 2021). Dementia, cancers, chronic respiratory diseases, autoimmune diseases and chronic kidney disease (CKD) are also enlisted in NCDs. They are generally reported as the leading cause of mortality in the world. Common and modifiable risk factors underlying most NCDs are tobacco, pollution, harmful use of alcohol, unhealthy diet, and physical inactivity (Peters et al., 2019).

Nowadays the IEMs are clinically well investigated and an earlier diagnosis is possible especially thanks to the expanded newborn screening. Therefore, an early treatment could guarantee an improvement of the *quod vitam* prognosis of these patients.

Life expectancy of these patients has markedly improved, allowing us to evaluate the possible onset of long-term comorbidities, such as NCDs, and trying to understand the causes with the aim of preventing them.

The lifelong special diet of people affected by IEMs may induce changes in the microbiota towards a pro-inflammatory profile with a consequent difference in the SCFAs production while the clinical expression of the disease itself could be directly responsible for altering the composition of the microbiota. Therefore, we could hypothesize a synergistic phenomenon, where two or more actors, like the dietary treatment, chronic use of drugs or supplementations, and the disease phenotype, may shape a typical microbiota signature, with a consequent dysbiotic status. Accumulating evidence indicates that an altered microbial community is correlated with a number of pathologies (Sekirov et al., 2010), ranging from metabolic (Sanmiguel et al., 2015; Tang et al., 2017) to immunologic and psychic diseases (Dinan and Cryan, 2017). Specifically, dysbiosis seems to contribute to the development of NCDs including type 2 diabetes, fatty liver disease and obesity (Tomasello et al., 2016). Hence, we need to address several questions: what long-term effects could have the observed dysbiosis in IEMs? Can a different intestinal microbiota increase the risk of NCDs in this population?

Up to date, there are still sparse studies in the literature that have tried to investigate these aspects in IEMs. Nevertheless, major findings are summarized in the following sections.

### Phenylketonuria

The quality of dietary carbohydrates of PKU children could directly affect the abundance of beneficial bacteria such as *Faecalibacterium*. In agreement with the data described so far, Fava et al. (2013) highlighted that a diet rich in carbohydrates with a high glycemic index leads to a reduction in the abundance of *Faecalibacterium prausnitzii* in subjects at risk of developing metabolic syndrome. Furthermore, some studies reported a reduction in the proportion of *Roseburia* spp. and *F. prausnitzii* in subjects with type 2 diabetes mellitus. These taxa are considered among major butyrate-producing bacteria and a reduced production of butyrate is reported to be associated with the development of insulin resistance both in humans and in the animal model (Qin et al., 2012; Karlsson et al., 2013). In PKU patients the different dietary pattern, focused on limiting Phe consumption, could be the cause of an unbalanced ratio Firmicutes/Bacteroidetes (Verduci et al., 2020). The balance of these two phyla ratio has an important role for obesity and higher body mass index (BMI), since a decreased ratio is associated with an increase in energy harvest, resulting in weight gain (Ley et al., 2005). Several studies reported that BMI and childhood obesity are influenced by ratio Firmicutes/Bacteroidetes (Indiani et al., 2018). Therefore, the modified microbiota of patients with PKU could potentially lead to a worse glycemic control, with a consequent insulin resistance, and weight gain (Verduci et al., 2020).

Since a different microbiota with a pro-inflammatory profile seems to predispose to NCDs in PKU subjects, probably due to a systemic pro-inflammatory state and an altered gut-liver axis, some studies reported a higher prevalence of overweight or increased fat mass (FM) in older subjects on dietary treatment, particularly in women (Acosta et al., 2003; Belanger-Quintana and Martinez-Pardo, 2011; Burrage et al., 2012; Aldamiz-Echevarria et al., 2014; Verduci et al., 2016; Sena et al., 2020). In particular, Scaglioni et al. investigated childhood overweight in a longitudinal observational study including 97 hyperphenylalaninemic children. They observed that 24.7% of children were overweight at the age of 8years with an earlier BMI rebound than non-overweight children and a higher BMI from the age of 1year (Scaglioni et al., 2004). An increase of triglyceride-glucose index (TyG index) in children with PKU compared to age and sex matched healthy controls was also observed (Moretti et al., 2017). The TyG index, calculated as Ln [TG (mg/dl)×glucose (mg/dl)/2], is considered a marker of low-grade inflammation and peripheral insulin resistance (Er et al., 2016). Moretti et al. (2017) showed a positive correlation between the TyG index and the glycemic load in PKU, that is higher than the normal values, strengthening the hypothesis of a possible link between the quality of carbohydrates and the predisposition to the development of metabolic disorders. In children with PKU an increase in the consumption of rapidly absorbed carbohydrates was observed, escaping the intestinal microbial fermentation with a consequent increase in the glycemic index and glycemic load (Bassanini et al., 2019).

Couce et al. studied the glucose metabolism in a cohort of 83 PKU and MHP patients and found increased fasting insulin levels in individuals with PKU compared to MHP subjects. Carbohydrate intolerance and insulin resistance were more evident in adults and in overweight patients. Patients treated with tetrahydrobiopterin (BH4) have been shown to have lower insulin and HOMA-IR levels, but the study was biased by the lack of a healthy control group (Couce et al., 2018).

A recent study showed that the cardiovascular phenotype of adult PKU patients is characterized by traditional cardiovascular risk factors, high levels of inflammatory and oxidative stress markers, endothelial dysfunction and vascular stiffness (Azabdaftari et al., 2019).

Morion Deon et al. reported an increased urinary oxidative stress parameter with a decreased urinary antioxidant capacity in PKU treated patients, associated with an increase of proinflammatory cytokines’ plasmatic levels, as interleukin-6 and interleukin-1. Specifically, urinary isoprostanes (oxidative metabolites, result of a damaged lipid oxidation) resulted positively correlated with interleukin-1 suggesting an enhanced inflammatory process in PKU patients, associated with lipid damage. In PKU patients it seems that both a restricted diet and the Phe metabolites, with an excessive production of reactive species, could impact on the oxidative stress (Deon et al., 2015). Moreover, Schulpis et al. (2005) reported a moderate hyperhomocysteinemia in PKU patients on a strict diet, with a possible endothelial activation and arteriopathy.

Overall, these data suggest that PKU patients may be vulnerable to a higher risk of obesity, insulin resistance and its complications, in a context of low-grade inflammation and enhanced oxidative stress with an increased cardiovascular risk, regardless of the cause.

### Glycogen Storage Disease-I

In hepatic GSDs, IBD, and liver disease are common features and they are part of the clinical expression of the pathology itself. Hepatocytes are sensitive to microbial products that may trigger an inflammatory immune response with systemic effects (Colonetti et al., 2019). GSD-I patients are at high risk for developing insulin resistance, traditionally attributed to nutritional “overtreatment” (Rossi et al., 2018). Especially in the past, in the attempt to avoid hypoglycemic crisis, there was a tendency to administer a high proportion of carbohydrates, sometimes exceeding the patient needs, with a consequent long-term risk of weight gain and hyperinsulinism with insulin resistance (Kishnani et al., 2014). Rossi et al. sustain that mitochondrial dysfunction has been implicated in the development of IR. The abnormalities in plasma acylcarnitines and urine organic acids found in GSD-I patients are indicative of a mitochondrial impairment, probably due to a possible distress on the intermediary metabolism as a consequence of the block of gluconeogenesis and glycogenolysis block. The oxidative stress seems to be related to higher insulin serum levels and other insulin resistance indexes, especially in GSD-Ia, and with altered lipid profile (Rossi et al., 2018).

Inflammatory bowel disease is a phenotypic expression of the disease in GSD-Ib, probably related to the neutrophil dysfunction, although it has also been described in GSD-Ia patients (Lawrence et al., 2017; Carnero-Gregorio et al., 2019).

Tomasello et al. reported the oxidative stress among the possible pathogenetic mechanisms of IBD. The increase of ROS could arise from an incomplete reduction of oxygen, related with dysbiosis, probably through a positive feedback mechanism, where oxidative stress prompts the gut inflammation exacerbating the ROS production and subsequent tissue damage (Tomasello et al., 2016).

### Other Diseases

In branched-chain amino acid disorders, i.e., organic acidurias (OA), and urea cycle disorders (UCD), few data are available in literature and the studies are mainly focused on body composition. Compared to the controls or reference values, a normal or increased FM has been reported in both children and adults affected by UCD and OA (Manoli, 2016; Evans et al., 2017).

An Italian observational study examined 17 adult UCD and OA patients on a low protein diet and detected a BMI >25 in 40% of the subjects analyzed, and an increased FM compared to normal values (Gugelmo et al., 2020). In UCD the few available data demonstrated an energy intake lower than the recommended value with a negative correlation between the percentage of FM and the total protein intake (Hook et al., 2016; Evans et al., 2017). Brambilla et al. (2019), in a more recent study, evaluated the resting energy expenditure in argininosuccinic aciduria (ASA) and in the other UCD, demonstrating that ASA had a resting energy expenditure (from indirect calorimetry, IC-REE) of 88% of the value predicted by the FAO and Harris-Benedict equations, whereas in the other UCD it was similar to the one expected. Low IC-REE was associated in ASA patients with increased prevalence of pathological waist circumference-to-height ratio, hypertension, hypertriglyceridemia and low HDL-cholesterol. No significant differences in body composition parameters were observed between the two groups. Definitely ASA patients have higher risk of obesity and increased cardiovascular risk. No data about microbiota in UCD are available, but it could be interesting to evaluate the presence of bacterial strains associated with proinflammatory patterns (Brambilla et al., 2019).

In OA branched-chain amino acid disorders and homocystinuria, an oxidative stress and mitochondrial dysfunction has been reported, as the effect of toxic metabolite accumulation, with significant multi-organ damage. The imbalanced ratio between the production and the adequate use of energy may provoke an inefficient cell metabolism as a feature in diseases like IEMs, underlining a link between hypertension, obesity, dyslipidemia and mitochondrial impairment (Ray and Mukherjee, 2021).

Although the prevalence of NCDs in IEMs is variable and the scientific evidence is still incomplete, we believe that IEMs deserve attention in this field. IEMs are predominantly monogenic disorders, but their phenotypic presentation is complex and heterogeneous as they can be the result of either a toxic accumulation of metabolites or deficiency in end products, going to impact different physiological systems (Kirby et al., 2020) also through an imbalance of oxidative state (Ray and Mukherjee, 2021).

Both the metabolic dysfunction and the dietary regimen are likely responsible for alterations in gut microbiome composition in IEMs patients. Indeed, dysbiosis, which determines a pro-inflammatory state alongside a condition of increased oxidative stress found in these patients, could be cumulative and concatenated risk factors. The contribution of gut microbiome in causing NCDs is still unclear, but of course an improvement of microbiota composition in anti-inflammatory and anti-oxidative direction can reduce the risk, since dysbiosis could move the clinical phenotype to further exacerbation as a potential patho-genetic mechanism, and it can also represent a preventive action in relation to NCDs (Kirby et al., 2020).

Hence, IEMs must be rethought and rearranged in a framework of complexity that cannot fail to take into account the intestinal microbiota and its dialog with other systems of the body through the gut-liver axis. There are still questions and hypotheses waiting to be confirmed or disapproved by future studies, such as: where in the pathophysiological chain gut dysbiosis is located? What its long-term effects may be? Can intestinal microbiota be a target in the treatment of IEMs? In our opinion, long-term studies designed in adult patients will be needed to better investigate eventual effects of dysbiosis in IEMs. This is the challenge for us in the scientific world, representing a completely open field of exploration that may allow a better care of the patient with IEMs.

## Potential Therapeutic Approaches Targeting the Gut-Liver Axis in Iems

In the light of data reported so far, we would like to underline the importance of the patient’s general health status, in the context of the periodical evaluation of patients affected by IEMs. It is essential to point out the crucial role of the nutritional status assessment, as well as of monitoring the key clinical and biochemical signs of an eventual onset of a metabolic comorbidity to prevent and reduce the NCDs risk and optimize the long-term health outcome in IEMs. It becomes necessary for a “best clinical practice patient-personalized” to promote a healthy food consumption, within the different dietary patterns indicated for the disease, and to encourage daily physical exercise. Therefore, we believe that an important step in the nutritional and clinical management of patients affected by IEMs could focus on improving dietary products and mixtures commonly used in IEMs, for example proposing a change in the quality of the low protein foods by a careful selection of starches and consequently modifying the quantity of soluble fibers, or paying attention to a the possible “overtreatment” with an excessive intake of carbohydrates to avoid hypoglycemia. Moreover, a supplementation with pro-, pre-, or postbiotics, within or without dietary products and mixtures, could be an important improvement of the quality of the diet.

### Functional Foods (Pre-, Pro-, Post-biotics)

Manipulating the microbiota composition is one of the potential implications of the growing evidence of microbiome alterations in IEMs. The so-called functional foods encompass a group of food products with a potentially positive effect on health (Altveş et al., 2020). Probiotics are “live microorganisms that administered in adequate quantities confer a health benefit on the host.” Prebiotics are non-digestible compounds whose positive effects are due to promoting the growth of a selected number of bacteria in the gut. Postbiotics are bacterial or metabolic products produced by probiotic microorganisms that have a biologic activity in the host (Tsilingiri et al., 2012; Tsilingiri and Rescigno, 2013).

To date, the role of functional foods in IEMs has been poorly investigated, in particular no evidences are available so far on a possible role of postbiotics in the context of IEMs and the majority of studies focus on the use of pre/probiotics in conditions which share physiopathological aspects, dietetic approach and/or secondary multiorgan involvement, paving the way to their possible application to IEMs. The potential impact of pre/probiotics treatment of PKU, the most common disorder among IEMs, found a greater development. MacDonald et al. (2011) investigated the possible beneficial effects of prebiotic oligosaccharides (scGOS/lcFOS), commonly present in breast milk, added in the amino acid mixture for PKU newborns. The supplemented AA mixture was able to keep *Bifidobacteria* and *Lactobacilli-Enterococci* levels at values comparable to healthy newborns. In the last decade, emphasis has been given to the role of GMP in the PKU diet, as naturally poor in Phe and more palatable than amino acid formulas. The gut microbiome plays an important role not only in amino acid metabolism, but also in carbohydrate and vitamin pathways, influencing the physiology of liver, brain and GI tract (Colonetti et al., 2018). This leads us to speculate that the microbiome modification may provide potential advantages in the management of many IEMs. Tang et al. (2017) reported on the synergistic effects of *Lactobacillus plantarum* S58 (LP.S58) and hull-less barley β-glucan (β-G) on lipid accumulation in mice fed with a high-fat diet. LP.S58 and β-G synergistically attenuated lipid accumulation by activating AMPK signaling and regulating the gut microbiota. In another recent study of Danhong et al. (2021), a supplement of probiotics is defined as a promising strategy for NAFLD and obesity treatment. Authors investigated the separated and combined effects of Bifidobacteria and resveratrol against obesity and NAFLD and concluded that a combination with a prebiotic substrate may improve the effects of probiotics. Probiotics, including *Lactobacillus rhamnosus* (LGG strain), have been shown to have several beneficial effects on the intestinal function by normalizing the dysbiotic microbiota (Resta-Lenert and Barrett, 2003; Bruzzese et al., 2004; Sartor, 2004; Ewaschuk and Dieleman, 2006; Versalovic, 2007; Marciano and Vajro, 2017). In particular, LGG has also been reported to reduce intestinal oxidative stress (Tao et al., 2006). Forsyth et al. (2009) showed that daily LGG treatment significantly improved severity of alcoholic steatohepatitis (ASH) and alcohol-induced gut leakiness, reduced markers of intestinal and liver oxidative stress and inflammation, and normalized the gut barrier function, preventing liver disease in a rat model of alcohol induced leaky gut and steatohepatitis. In pediatric patients with NAFLD, the subsequent changes in gut microbiome composition allowed a NAFLD-linked microbiota profiling (Del Chierico et al., 2017) and to design a tailored probiotics treatment (Putignani et al., 2016; Nobili et al., 2018, 2019). Rodríguez-Cerdeira et al. (2019) showed how the administration of a mixture of *Lactobacilli*, *Bifidobacteria*, and *Streptococcus thermophilus* in a young GSD-I woman can modify gut microbiota and improve the patient’s quality of life in terms of ameliorating irritable bowel symptoms. Regular administration of the probiotic mixture led to an increase of Bacteroidetes, *Clostridium leptum* and *Eubacterium* and to a decrease in *Enterobacteriaceae* (*Escherichia*, *Klebsiella*, *Proteus*; Carnero-Gregorio et al., 2019). In OA prebiotics could also play an important role. Burlina et al. supposed that prebiotics that lower pH in the gut microbiota environment could potentially decrease propionate production so stimulating the activity of lactate and the predominant acetate-producing species could reduce propionate production. Studies in healthy adults have shown that FOS alone stimulates subsequent cross-feeding, i.e., metabolism of lactate to butyrate (Belenguer et al., 2006; Rios-Covian et al., 2015). As the properties of butyrate are closer to propionate, in the human gut microbiota, this finding needs careful consideration in the development of prebiotic strategies for the management of PA/MMA. Other studies in healthy infants have shown that prebiotics containing GOS/FOS (9:1) stimulate acetate and lactate production, while suppressing propionate and butyrate production (Oozeer et al., 2013). A controlled study in healthy infants showed that GOS/FOS mixture added to infant formula stimulated the growth of *Bifidobacteria* and increased the overall metabolic activity of intestinal microbiota, resulting in higher acetate and lower propionate levels (Knol et al., 2005). By lowering pH, the prebiotic mixture can make the gut less hospitable to pathogens and decrease production of propionate (Belenguer et al., 2007). Therefore, the efficacy of any potential approach to reduce propionate production, such as the prebiotic administration of GOS and FOS, should be tested in patients with PA/MMA.

There is no direct evidence of the possible role of functional foods in lysosomal storage diseases (LSD). Recent studies show the role of microbiome modulation in slowing the progression of end stage-renal failure, a complication and frequent cause of death of Fabry disease. Vaziri et al. (2013) demonstrated the proliferation of dysbiotic bacteria in patients with CKD, which translocate in the systemic circulation through the impaired intestinal barrier. A recent randomized, double-blind, placebo-controlled trial on CKD patients, following a 6-month probiotic therapy, reported a significant reduction in the serum levels of endotoxin and pro-inflammatory cytokines, an increase of the anti-inflammatory cytokine IL-10, and a preservation of residual kidney function (Wang et al., 2019). The potential role of prebiotic use to slow down the CKD in OA such as methylmalonicacidemia, should take into account a possible effect in increasing propionic acid production and should be limited to non-propiogenic compounds (Burlina et al., 2018). The same mechanism of pathological translocation of commensal bacteria has been described also in several myopathies (Du Preez et al., 2018). The consequent altered immune response and systemic chronic inflammation, a common feature of inherited metabolic myopathies such as Pompe disease, can speculate a role of manipulating the altered microbiome also in this condition. Many studies suggest that increasing gut bacterial SCFAs production may positively affect skeletal muscle mass and physical function in humans (Lustgarten, 2019). In terms of bacterial species that may positively impact muscle mass, *Lactobacillus casei* or *Bifidobacterium longum* demonstrated to increase the muscle mass/body weight ratio (Ni et al., 2019). Overall, the use of functional foods in IEMs may represent a potential approach to all the above-mentioned conditions. As the administration of the probiotic *Lactobacillus reuteri*, engineered to express a phenylalanine lyase gene from the cyanobacteria *Anabaena variabilis*, demonstrated its efficacy in reducing Phe levels in PKU mice, representing a potential safe approach to PKU patients, the creation of genetically modified probiotics able to normalize defective metabolic pathways or controlling multiorgan complications, may represent a future therapeutic approach also to many other IEMs.

## Conclusion

The next challenges of current microbiome research should be to identify the mechanisms by which metabolic exchanges drive the diet-microbiome-pathophysiology interactions in IEMs, characterizing the gut microbiota in IEM patients beyond DNA-based composition analysis and incorporate other “omics” technologies, such as (meta)transcriptomics, (meta)proteomics, and (meta)metabolomics. The application of shotgun approaches in metagenomics pipelines will substantially contribute on the deep characterization at species and strain levels of microorganisms, particularly involved in metabolism regulation, such as SCFA and ethanol producers.

Furthermore, a more specialized microbiomics, based on shallow shotgun and trans kingdom metagenomics will improve the clinical microbiology dedicated to the understanding of microbiome in metabolic diseases, including not only bacteriome, but also virome, protozoa, and metazoan (i.e., parasitome) reservoirs, including their metabolic relationships. The new approach will be based on an “agnostic” view to characterize, through the different taxa, all possible microorganisms.

It will be necessary to turn attention to the intestinal ecosystem as a set of microbial metabolic interactions rather than to single isolated biotic factors (Burlina et al., 2018) in the framework of clinical DSSs driven by advanced bioinformatics and artificial intelligence-based pipelines and to study their possible long effects in IEMs.

Finally, modelling the intestinal microbiota through non-pharmacological nutritional treatments, based on a healthy diet and the use of specific pre-, pro-, and postbiotics, may represent an innovative approach of “precision medicine” in microbiomics focused on restoring microbiota balance in IEMs, thus significantly improving the health status of these subjects.

## Author Contributions

MC and EV conceptualized and reviewed the article. CM and SP collected all the data. CM, SP, GBa, JZ, CB, and AT drafted the manuscript. EB and LP critically revised the manuscript. CV, GBi, and AB gave the final approval. All authors contributed to the article and approved the submitted version.

## Conflict of Interest

The authors declare that the research was conducted in the absence of any commercial or financial relationships that could be construed as a potential conflict of interest.

## Publisher’s Note

All claims expressed in this article are solely those of the authors and do not necessarily represent those of their affiliated organizations, or those of the publisher, the editors and the reviewers. Any product that may be evaluated in this article, or claim that may be made by its manufacturer, is not guaranteed or endorsed by the publisher.
