# Supplementary material for: Dysbiosis, Host Metabolism, and Non-communicable Diseases: Trialogue in the Inborn Errors of Metabolism
Source: Front Physiol. 2021 Sep 6;12:716520. doi: 10.3389/fphys.2021.716520 (PMC8475650; doi:10.3389/fphys.2021.716520)
Supplement: Supplementary file 1 [file Data_Sheet_1.docx]

Supplementary Material

**Table S1.** Characteristics of gut microbiota profiles studied in PKU.

| **Reference** | **Cohort** | **Mean Age (years)** | **Methods and Platforms** | **Results**  **(PKU vs Controls)** |
| --- | --- | --- | --- | --- |
| Mancilla et al., 2021 | 11PKU  21 HC^a^ | 33.0 ± 1.98  29.0 ± 3.07 | 16S rRNA sequencing:  V4 hypervariable region;  MiSeq Illumina platform;  VSEARCH v2.8.1 and  EzBiocloud | ↑ *Clostridium* spp.  ↓ *Faecalibacterium* and *Blautia* spp. |
| Bassanini et al., 2019 | 21 PKU  21 MHP^d^ | 8.0 ± 3.4  10.0 ± 3.5 | 16S rRNA sequencing:  V3-V4 hypervariable regions; MiSeq Illumina platform;  Version 13.8 | ↓α-diversity^b^  β- diversity^c^ (*p*≤ 0.05)  ↑*Lachnospiraceae (other), Blautia* and *Clostridium* spp.  ↓*Ruminococcaceae (other), Faecalibacterium* and *Dialister* spp. |
|  |  |  | SCFA^e^ quantification: Gas chromatography | ↓Total SCFA and butyrate (in feces) |
|  |  |  |  |  |
| Pinheiro de Oliveira et al., 2016 | 8 PKU  10 HC^a^ | 4.24 ± 1.74  6.06 ± 1.78 | 16S rRNA sequencing:  V4 hypervariable regions;  IonTorrent platform;  Version 13.8 | ↓α-diversity^b^  β- diversity^c^ (*p*≤ 0.003)  ↑ Bacteroidetes, Verrucomicrobia  ↓ Firmicutes  ↑ Peptostreptococcaceae, *Akkermansia,Prevotella* spp.  ↓*Coprococcus, Dorea, Lachnospira, Odoribacter, Ruminococcus* and *Veillonella* |

^a^HC, healthy controls; ^b^α-diversity, the diversity in the bacterial composition within each sample; ^c^β- diversity, the diversity between sample groups; ^d^MHP, mild hyperphenylalaninemia patients as controls; ^e^SCFA, short-chain fatty acids.

**Table S2.** Characteristics of gut microbiota profiles studied in GSD.

| **Reference** | **Cohort** | **Mean Age (years)** | **Methods and Platforms** | **Results**  **(GSD vs HCa)** |
| --- | --- | --- | --- | --- |
| Ceccarini et al., 2020 | 9 GSD  (Ia=4, Ib=5)  12 HC^a^ | 27.7 ± 12.5  4.7 ± 7.9 | 16S rRNA sequencing:  V3-V4 hypervariable region; MiSeq Illumina platform; Greengenes Version 13.8  SCFA^d^ quantification:  Gas chromatography | ↓α-diversity^b^  β- diversity^c^ (*p*≤ 0.004)  ↑ Proteobacteria  ↑*Enterobacteriaceae* and *Veillonellaceae*  ↓*Ruminococcaceae*  ↑*Escherichia* spp.  ↓*Ruminococcus*, *Faecalibacterium* and *Oscillospira* spp.  ↑ Total SCFA, acetate and propionate (in feces) |
| Colonetti et al., 2019 | 24 GSD  (Ia=15, Ib=5, III=1, IXα=3)  16 HC^a^ | 12  12.5 | 16S rRNA sequencing:  V4 hypervariable regions;  IonTorrent platform;  Version 13.8 | ↓α-diversity^b^  β- diversity^c^ (*p*≤ 0.003)  ↑ Proteobacteria and Actinobacteria  ↑*Lactobacillus* and *Escherichia/Shigella*  ↓*Alistipes, Faecalibacterium* and *Roseburia* |

^a^HC, healthy controls; ^b^α-diversity, the diversity in the bacterial composition within each sample; ^c^β- diversity, the diversity between sample groups; ^d^SCFA, short-chain fatty acids.
